# Development of Double-Film Composite Food Packaging with UV Protection and Microbial Protection for Cherry Preservation

**DOI:** 10.3390/foods14132283

**Published:** 2025-06-27

**Authors:** Han Wang, Yanjing Liao, Guida Zhu, Longwen Wang, Zihan Chen, Xue Li, Chao Wang, Jing Yu, Ping Song

**Affiliations:** 1 School of Food Science and Pharmaceutical Engineering, Nanjing Normal University, Nanjing 210023, China; 2 Science Center for Future Food, Jiangnan University, Wuxi 214122, China

**Keywords:** food packaging, cherry, mechanical properties, antibacterial properties

## Abstract

This study develops a novel dual-layer chitosan (CS)/pectin film incorporating grape skin anthocyanin extract (GSAE) and lignin to address critical limitations in cherry preservation. Unlike traditional methods that leave harmful residues, this bilayer design separately integrates functional components: GSAE for targeted antioxidant/antibacterial action and lignin for ultraviolet (UV) blocking. This targeted incorporation enables synergistic performance unattainable with single-layer or conventional approaches. The films, fabricated with lignin concentrations from 1% to 15% (*w*/*v*), demonstrated excellent mechanical integrity (assessed with structural characterization), optimized gas barrier performance, and effective UV attenuation (achieved via lignin incorporation). Antibacterial analyses confirmed substantial inhibition against *Staphylococcus aureus* and *Escherichia coli*. Crucially, cherry preservation tests showed that the 15% lignin film (PG/CL15%) reduced weight loss, preserved firmness, and extended shelf life by 8 days—a significant quantitative improvement over uncoated fruit. Structural characterization (TGA, FT-IR, and XRD) verified successful GSAE/lignin embedding via hydrogen bonding. Beyond cherries, this dual-layer, bio-based design offers a promising template for the active packaging of other perishable produce sensitive to oxidation, microbial spoilage, and UV degradation, which enhances its industrial relevance.

## 1. Introduction

Cherry fruits are juicy and thick, and rich in nutrients [1]. Rich in natural health-care ingredients like anthocyanins and quercetin, they offer functions such as antioxidation, regulating physiological rhythms, and improving sleep quality and brain health [2]. Global cherry consumption has shown an overall upward trend. During cold chain logistics operations, cherries demonstrate heightened susceptibility to microbial spoilage and oxidative degradation, resulting in substantial resource depletion and economic attrition [3]. Current cherry preservation techniques primarily involve chemical preservatives, wax coatings, and plastic film packaging. However, these methods may leave chemical residues, increasing toxicological risks. Therefore, the development of healthy and sustainable food packaging has become imperative [4].

Currently, bio-based food packaging materials are increasingly becoming a focal point of research due to their exceptional preservation potential, with polysaccharide-based packaging materials receiving particular attention [5]. These materials can encapsulate natural active substances to enhance their functionality. In polysaccharide-based packaging systems, chitosan (CS) and pectin have found extensive use in the preservation of fruits and vegetables. This is attributed to their excellent loading capacity and favorable physicochemical properties [6]. However, packaging systems based solely on CS or pectin demonstrate limited antibacterial inhibition effects, failing to meet preservation requirements [5]. In prior research, Bi et al. incorporated proanthocyanidins into chitosan, which improved the tensile strength and thermal stability of the film [7]. Fu et al. successfully developed CS/pectin bilayer films by utilizing chitosan (CS) and edible citrus pectin as the film-forming matrix materials [8]. While these CS/pectin films show excellent gas barrier and mechanical properties, improvements are still required in UV shielding capacity, microbial inhibition, and antioxidant activity efficiency. Thus, the exploration of CS/pectin-based multifunctional active packaging incorporating bioactive components warrants additional research.

Therefore, integrating substances with strong antioxidant and antibacterial capabilities, as well as stabilizing and UV-protective properties, into a chitosan/pectin matrix presents a promising strategy to enhance the functionality and stability of bio-based food packaging materials. Grape skin anthocyanin extract (GSAE) is a natural anthocyanin-based pigment which can effectively scavenge DPPH free radicals, inhibit oxidase activity, and suppress microbial growth [8,9]. However, anthocyanins are particularly prone to photodegradation [10,11]. This degradation limits the application scope of anthocyanins and the storage period of related products. Consequently, the incorporation of a stabilizing protective component becomes essential. Lignin, which ranks as the second most plentiful natural aromatic polymer in nature, possesses exceptional functional properties, including ultraviolet absorption, and thermal stability [12]. Its environmental compatibility, cost-effectiveness, and accessibility further establish it as a widely recognized green functional material [13]. The addition of lignin can improve thermal stability in film materials [14,15]. Therefore, lignin incorporation significantly improves the stability of GSAE.

In this study, CS/pectin bilayer films were prepared by layer-by-layer casting technology, and multifunctional active packaging films with strong antioxidant and antibacterial activities were successfully developed by the hierarchical addition of grape skin anthocyanin extract (GSAE) and lignin. The mechanical strength, ultraviolet-blocking capability, gas barrier properties, and antibacterial activity against various foodborne pathogens of the bilayer films were comprehensively evaluated. Furthermore, the practical effectiveness of the films was validated through cherry preservation experiments. These research findings provide innovative approaches to the development of sustainable composite active packaging systems.

## 2. Materials and Methods

### 2.1. Materials

Chitosan was obtained from Shanghai MacLean Biochemical Technology Co., Ltd., Shanghai, China. Citrus pectin was obtained from Yantai Andeli Pectin Co., Ltd., Yantai, China. Grape pomace was supplied by the laboratory, and wine lees were sourced from Sichuan Luzhou Laojiao Co., Ltd., Luzhou, China. Fresh cherries were procured from Baiguoyuan Fruit Industry, located in Nanjing, Jiangsu Province, China. All chemical reagents were obtained from Sinopharm Chemical Reagent Co., Ltd., in Shanghai, China, including methanol (HPLC grade, ≥99.9%), ethanol (absolute, ≥99.8%), glacial acetic acid (AR grade), glycerol (USP grade), anhydrous calcium chloride (≥93%), sodium chloride (BioUltra grade), and potassium hydroxide pellets (ACS reagent, ≥85%). Additionally, the deoxidizer was purchased from Jiaxing Ganjiang New Materials Co., Ltd., Jiaxing, China.

### 2.2. Extraction of GSAE and Lignin

A grape skin residue sample (1.00 ± 0.01 g) was extracted using an ethanol–water solution (75% *v*/*v*, neutral pH) with the following parameters: a solid-to-liquid ratio of 1:15 *w*/*v*, constant-temperature maintenance at 60 °C, and an extraction duration of 45 min. The resultant extract was centrifuged at 4000× *g* for 15 min at 25 °C to separate insoluble particulate matter, yielding the supernatant, which served as the anthocyanin sample [16].

The lignin extraction experiment was conducted according to the methodology established by Rizg, W.Y. [17]. The alkaline solution containing dissolved distiller’s grains was taken, and dilute H_2_SO_4_ was slowly added dropwise to pH 2–3 under stirring and heating (50–70 °C). The mixture was maintained at this pH and continuously stirred for 15–30 min for ripening. Subsequently, the suspension was transferred to a centrifuge tube and centrifuged at 4000–8000 rpm for 10–20 min, with the supernatant discarded. The precipitate was resuspended in acidic water (pH ≈ 3) and centrifugally washed 2–4 times, repeating the centrifugation until the supernatant became nearly colorless. Finally, the precipitate was dried in an oven at 45–60 °C for 24–48 h to obtain tan lignin powder.

### 2.3. Film Preparation

Double-layer films incorporating lignin and grape seed antioxidant extract (GSAE) were fabricated using a layer-by-layer casting technique. Chitosan (CS) film-forming solutions (FFSs) were created as follows: First, 2 g of CS was dissolved in a 2% (*v*/*v*) acetic acid aqueous solution. Subsequently, varying amounts of lignin (0.02, 0.1, 0.2, and 0.3 g) were individually incorporated into the solution. This process resulted in the formation of a CS–lignin FFS series (CL1–CL15%), with the solutions having lignin concentrations of 1%, 5%, 10%, and 15% (*w*/*v*). A pectin solution (PG-FFS) was formulated by dissolving of 2 g of citrus pectin in 100 mL of DI water, adding 0.5 g of GSAE, and stirring continuously for 12 h at 25 °C. The pectin solution without GSAE addition is referred to as P-FFS. All solutions were supplemented with 25% (*w*/*w*) glycerol as a plasticizer and ultrasonically degassed for 30 min prior to use. Initially, 20 g of CL-FFS was transferred into a mold and thermally cured at 45 °C, followed by casting a 20 g layer of PG-FFS for secondary curing (45 °C, 14 h) to construct the bilayer structure. The experimental samples (P/C, PG/C, and PG/CL1–CL15%) were subjected to drying in a desiccator with constant humidity at 25 °C and 50% relative humidity for at least 48 h before performance characterization. The mold with dimensions of 10 × 10 × 2 cm was used. A scraper was employed to level each layer of the film during preparation to control the thickness. After the film was formed, a knife was used to separate the film from the mold.

### 2.4. Analysis of Film Properties

#### 2.4.1. Color

Chromatic analysis was conducted using a calibrated colorimeter following CIE Lab* color space specifications. Instrument calibration was carried out using a certified white reference tile (L* = 93.48 ± 0.12, a* = −0.65 ± 0.03, b* = 1.91 ± 0.08). The color values of L (lightness), a (red/green), and b (yellow/blue) were applied. The total color difference (ΔE) and the whiteness index (WI) were determined utilizing Equations (1) and (2):

ΔE = [(L* − L)2 +(a* − a)^2^ +(b* − b)^2^]^0.5^ (1)

WI = 100 − [(100 − L)^2^ + a^2^ + b^2^ ]^0.5^ (2)

#### 2.4.2. Optical Properties

The film specimen was secured in quartz cuvettes for UV–Vis spectral analysis (200–800 nm) using a spectrophotometer (UV-4802, UNICO, Franksville, WI, USA). Absorbance at λ = 600 nm was utilized for opacity determination via Equation (3) [18]:

Opacity = A600/d(3)

where A600 denotes the absorbance measured at a wavelength of 600 nm, while d represents the average thickness of the film, expressed in millimeters.

#### 2.4.3. Mechanical Properties

The mechanical properties of the film were tested according to the Fu X method [4]. The tensile strength (TS) and elongation at break (EAB) of the film samples (80 mm × 10 mm) were tested using a Brookfield CT3 texture analyzer (New York, NY, USA) in tensile mode. The samples were measured with a 50 mm grip separation and a stretching rate of 10.0 mm/min.

#### 2.4.4. Water Contact Angle

The surface hydrophobicity was quantified by static contact angle measurement, where a 10 μL droplet of deionized water was deposited onto the thin film and measured under ambient conditions by using an OCA25 optical contact angle goniometer (Dataphysics, Filderstadt, Germany) [19].

#### 2.4.5. Microstructure

Surface topography characterization was performed using atomic force microscopy (Bruker Dimension Icon, Saarbrücken, Germany). Average roughness (Ra) and root mean square roughness (Rq) were determined using Nanoscope Analysis v1.8 software in PeakForce Tapping Mode (scan size: 5 × 5 μm^2^; resolution: 512 × 512 pixels) [20].

#### 2.4.6. Gas Barrier Properties

The film sample was placed into a permeation cell containing 12.00 g of anhydrous calcium chloride desiccant. The samples were incubated for 48 h in a constant-temperature incubator (model LHS-100CL, Shanghai Yiheng Scientific Instrument Co., Ltd., Shanghai, China) maintained at 25.0 °C and 75% relative humidity. Mass measurements were conducted both before and after the incubation process. WVP values were calculated using Equation (4) [21,22]:

WVP = (ΔM × d)/(A × t × ΔP)(4)

where ΔM refers to the mass increase measured in kilograms (kg), d indicates the average thickness of the film expressed in meters (m), A denotes the effective area for water vapor transmission (m^2^), t signifies the duration (s), and ΔP signifies the difference in water vapor pressure across the film, measured in pascals (Pa).

#### 2.4.7. Antioxidant Activity

DPPH radical scavenging capacity was assessed via a modified method. Precisely weighed film aliquots (4–20 mg (P/C, PG/C, PG/CL1%, PG/CL5%, PG/CL10%, and PG/CL15%) underwent aqueous solubilization (1 mL water) followed by a reaction with 3 mL of 1 mM DPPH methanolic solution. After 30 min of dark incubation (37 °C thermostatic water bath), the mixtures were centrifuged (6076× *g*, 20 min, 4 °C). Absorbance at λ = 517 nm was measured using a SpectraMax M5 microplate reader. Radical scavenging efficiency was calculated using Equation (5) [23,24]:

DPPH scavenging effect (%) = [(A_0_ − A_1_)/A_0_] × 100% (5)

where A_0_ and A_1_ represent the absorbance values of ABTS for the control sample (without film) and the sample with film, respectively.

The test method is based on Jiang’s method [25]: First, 7 mM ABTS solution and 2.45 mM potassium persulfate solution were mixed in a 1:1 (*v*/*v*) ratio and reacted for 12 h. The mixture was then diluted with ethanol to adjust the absorbance to 0.70 ± 0.02 at 734 nm. For sample preparation, 4, 8, 12, 16, or 20 mg of each film was dissolved in 1 mL of deionized water, followed by mixing with 3 mL of the ABTS solution. The reaction was carried out in the dark for 30 min, after which the mixture was centrifuged at 6076× *g* for 20 min at room temperature. The absorbance of the supernatant was measured at 734 nm. The ABTS radical scavenging activity of the film was determined by Equation (6):

ABTS scavenging effect (%) = [(A_0_ − A_1_)/A_0_] × 100% (6)

where A_0_ and A_1_ are the ABTS absorbance values of the control (no film) and the film, respectively.

#### 2.4.8. X-Ray Diffraction (XRD)

XRD testing was based on the Zhao Y method [5]. The XRD analysis of the film sample was performed using a Bruker D8 Advance X-ray diffractometer (Bremen, Germany) at 40 kV and 30 mA, with the 2θ scanning range set from 5° to 45°.

#### 2.4.9. Fourier Transform Infrared (FT-IR) Spectroscopy

FT-IR testing was based on the Ghadetaj method [26]. The FT-IR spectra of the film samples were acquired using a Nicolet Nexus 410 spectrometer (East Lyme, CT, USA). The spectral analysis covered a wavenumber range of 4000–400 cm^−1^ at a resolution of 4 cm^−1^.

#### 2.4.10. Thermogravimetric Analysis (TGA)

Thermogravimetric analysis (TGA) was carried out with a Netzsch STA 449 F3 Jupiter analyzer (Netzsch-Gerätebau GmbH, Selb, Germany) under a dynamic nitrogen atmosphere with a flow rate of 50 mL·min^−1^. The samples underwent programmed heating from 25 to 600 °C at 10 °C·min^−1^, with simultaneous derivative thermogravimetry (DTG) profiling to assess thermal degradation kinetics across the composite film series [27].

#### 2.4.11. Antimicrobial Characteristics

*Staphylococcus aureus* (*S. aureus*) and *Escherichia coli* (*E. coli*) were obtained from the laboratory collection. The agar diffusion method was utilized to evaluate the antibacterial efficacy of the films. Bacterial cultures were incubated in nutrient broth at 37 °C for 24 h. Subsequently, 0.1 mL of standardized inoculum (1 × 10^6^ CFU/mL) was aseptically spread onto Luria–Bertani (LB) agar plates [28]. Sterile film discs (10 mm diameter) were positioned on LB agar plates and incubated under standard conditions (37 °C, 24 h). Inhibition zone diameters were quantitatively measured using a digital micrometer (Mitutoyo, ±0.01 mm accuracy). Experimental groups included PG/C and PG/CL15% bilayer composites, with P/C bilayer films serving as the negative control. UV-C irradiation (253.7 nm wavelength, 15 W power) was applied through an isometric exposure protocol (30 cm vertical distance from source), with antimicrobial efficacy assessed via agar diffusion assays at specified irradiation intervals (0, 1, 2, and 3 h) [29].

#### 2.4.12. Application of Double-Layer Composite Active Film in Cherry Preservation

Cherries selected through cold chain transportation exhibited uniform maturity, absence of mechanical damage, and standardized fruit diameter. The experimental groups consisted of an uncoated control (CK), P/C bilayer film, PG/C composite film, and PG/CL15% active film. Sequential dip-coating was performed in 2% (*w*/*v*) PG solution and CL solution (prepared with 2% acetic acid), each for 30 s, followed by forced air drying (25 °C, 15 min, 0.5 m/s airflow) to form continuous bilayer coatings. The coated cherries were stored under ambient conditions for 8 days, with mass loss being quantified gravimetrically using a Sartorius CPA225D analytical balance (±0.1 mg accuracy) according to Equation (7) [29]:

Weight loss (%) = [(W_0_ − W_1_)/W_0_] × 100% (7)

In this context, W_0_ denotes the initial weight of the cherry fruit, while W_1_ is the weight of the cherry fruit at different storage intervals. Fruit firmness was determined through texture profile analysis using a Brookfield CT3 texture analyzer equipped with a TA-39 cylindrical probe. Standardized puncture tests were conducted at the equatorial plane under controlled parameters: 7 mm penetration depth at 0.5 mm/s crosshead speed (ASTM D882), with maximum force (N) recorded. For physicochemical analyses, juice aliquots were obtained through centrifugal separation (6076× *g*, 15 min, 4 °C; Eppendorf 5810R). Soluble solids content (SSC, °Brix) was quantified using a digital refractometer (Atago PAL-1) following AOAC 932.12, while titratable acidity was measured via potentiometric titration (AOAC 942.15) using 20 μL clarified supernatant [30].

### 2.5. Statistical Analysis

All tests on the films were repeated three times. Statistical analyses were conducted using SPSS 20.0 software (Chicago, IL, USA). All data were analyzed through one-way analysis of variance (ANOVA) with Duncan’s test and are presented as means ± standard deviations.

## 3. Results and Discussion

### 3.1. Optical Characteristics of Bilayer Films

The chromatic characteristics of food packaging surfaces critically mediate consumer visual perception and product acceptance extent. Visual variations among packaging materials are principally manifested through measurable differences in color values and ultraviolet spectral transmittance [31]. Color difference analysis revealed no significant variations in color values between PG/C films containing GSAE and P/C control films (Table 1). Following the incorporation of lignin, the composite film exhibited a decreased L value alongside slight increases in a and b values. The reduced L value correlates with diminished film brightness at higher lignin concentrations, while elevated a and b values indicate a chromatic shift from colorless to reddish-brown. This chromatic transition likely originates from the inherent reddish-brown color of lignin in the free film solution (FFS). Film opacity, quantified through absorbance measurements at 600 nm, demonstrated significantly increased values in PG/CL films (*p* < 0.05), which corresponded with reduced visual clarity. Increasing lignin concentration resulted in a concentration-dependent increase in film opacity, primarily due to two mechanisms: (1) the gradual darkening of the lignin film-forming solution from colorless to yellowish-brown and (2) enhanced molecular interactions among lignin, pectin, and chitosan (CS) components, which promote a tighter packing of polymer chains and reduce light permeability [32]. The obtained composite films, similar to other lignin-containing packaging films, can increase opacity [4,33].

### 3.2. The Light-Shielding Properties of the Films

**Figure 1:**
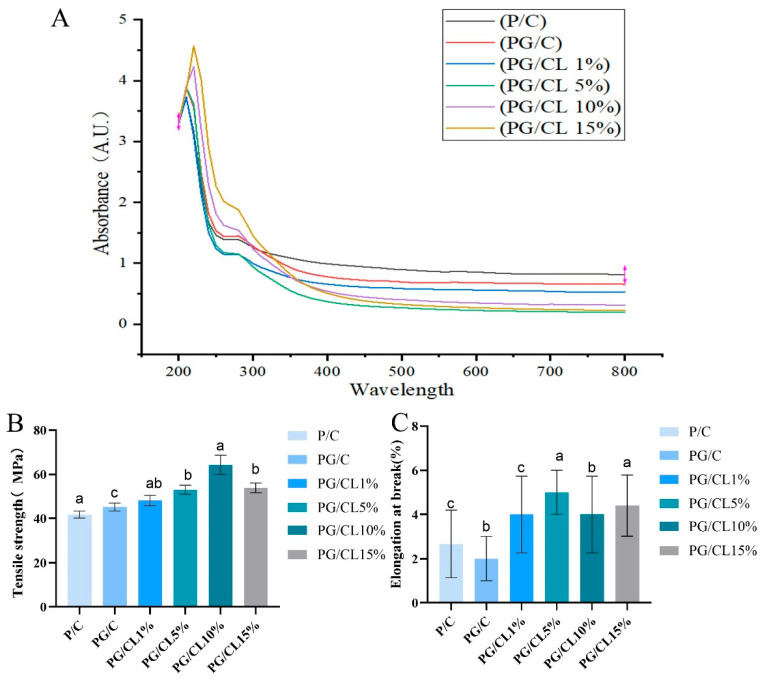
Ultraviolet–visible transmittance (**A**) and mechanical properties (**B**,**C**). Lowercase letters indicate significant differences in the properties of different films (*p* < 0.05).

Ultraviolet (UV) radiation (200–380 nm) induces oxidative food deterioration, necessitating a rigorous evaluation of UV-blocking capacity when developing cherry preservation packaging. The pectin/CS bilayer film functionalized with GSAE and lignin exhibited superior light absorption capacity relative to P/C controls (Figure 1A and Figures S1–S6). Notably, GSAE incorporation enhanced the PG/C film’s light absorption capacity across UV wavelengths. Subsequent lignin addition resulted in markedly enhanced light absorption capacity, particularly within the critical 200–380 nm range. However, in the visible light range of 380–800 nm, the absorbance value of the PG/CL15% film decreased significantly, which is different from the results of previous studies [12]. Research findings have demonstrated that lignin, as a biopolymeric substance, exhibits a three-dimensionally cross-linked architecture formed through the interconnection of three distinct phenylpropane-derived monomeric components: p-hydroxyphenyl (H), guaiacyl (G), and syringyl (S) units. These structural elements are chemically bonded through both ether linkages and direct carbon–carbon covalent bonds, creating the complex macromolecular framework characteristic of this natural polymer [34]. The aromatic rings in lignin interact with active groups (such as methoxy, phenolic, carbonyl, and vinyl groups) to confer UV resistance to lignin. Therefore, in this study, chitosan–lignin composite films are proposed as biological defense substances that absorb UV radiation. The phenolic hydroxyl groups and chromophores inherent in the lignin structure enable the specific absorption of ultraviolet (UV) rays. Upon UV irradiation, the phenylpropane units in lignin undergo conversion into quinone structures, thereby reducing or even blocking UV transmission to achieve shielding effects. Based on spectral superposition theory, it is predicted that the photodegradation process of GSAE will be regulated by lignin [4,35]. Additionally, the obtained composite films exhibit excellent UV-absorption properties, which are consistent with those of other lignin-containing packaging films. With the protection of lignin, GSAE is expected to maintain sustained antioxidant activity, making it suitable for use in cherry preservation packaging.

### 3.3. Mechanical Properties of Films

The packaging material must exhibit sufficient mechanical strength and flexibility to withstand external pressures, ensuring the integrity of the food during storage. After incorporating lignin into the active film matrix, the tensile strength (TS) significantly increased from 49.94 MPa to 71.64 MPa, while elongation at break (EAB) remained stable between 2.54% and 3.71% (Figure 1B,C). At lignin concentrations of 5–15%, TS showed a statistically significant improvement (*p* < 0.05), whereas EAB exhibited no notable change (*p* > 0.05). The gradual addition of lignin promoted new intermolecular hydrogen bonds between pectin and CS. These polyphenolic interactions with the matrix components enhanced structural integrity and improved mechanical tensile resistance. The findings demonstrate that lignin concentration significantly influences the tensile properties of PG/CL films [34]. We also observed a slight decrease in the tensile strength of PG/CL15% films, which may be due to the interference of excessive lignin with the formation of intermolecular hydrogen bonds during the drying process of the films, resulting in a decrease in tensile strength of the composite films [36]. The reduction in the mechanical properties of films due to the addition of lignin has been reported in previous studies, but the mechanism still needs to be further investigated [37,38].

### 3.4. Measurement of Hydrophobicity of Films

**Figure 2:**
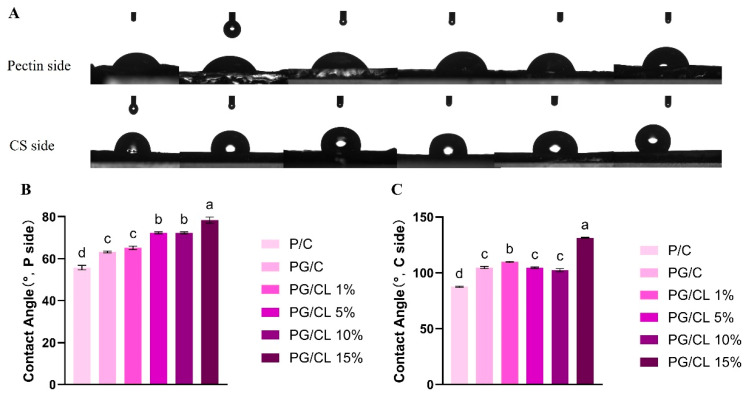
The water contact angles of the bilayer films are examined, with the pectin side representing the inner surface of the film and the chitosan (CS) side constituting the outer surface. The contact angle image of the film surface (**A**). The measured contact angle values of the film (**B**,**C**). Lowercase letters indicate significant differences in the properties of different films (*p* < 0.05).

The water contact angle magnitude depends on the interfacial interaction between water and the membrane surface upon contact. Materials exhibiting contact angles <90° are considered hydrophilic due to their strong water affinity. For pure pectin films, contact angles ranging from 56.47° to 78.36° were observed on the pectin surface. Notably, GSAE-incorporated pectin films showed increased contact angles, with PG/CL10% and PG/CL15% demonstrating values of 82.30° and 80.97°, respectively (Figure 2). This apparent hydrophobicity enhancement paradoxically occurs despite the film’s abundant -OH and -COOH groups, which can be attributed to altered surface characteristics. Furthermore, surface roughness significantly influences the film’s contact angle measurements, and it has been proved that the hydrophilicity of the rougher film surface is also lower [39]. In combination with the increase in the pectin-side roughness in AFM, there is no statistically significant difference observed in the values of the film contact angle on the surface of the film on the PG side (*p* > 0.05). The membrane on the CL side becomes hydrophobic and increases with the increase in lignin, probably because lignin becomes hydrophobic. The hydrophilic moieties (e.g., hydroxyl groups) within anthocyanin molecules establish hydrogen bonding and electrostatic interactions with lignin-based films, processes that facilitate molecular reorientation and diminish the surface exposure of hydrophilic functional groups—thereby resulting in elevated contact angle measurements [33]. Lignin contains a large number of hydrophobic groups, and the article by Yu et al. [40] also confirms this finding. The differential surface hydrophilicity between packaging layers facilitates timely water vapor release generated through cherry fruit respiration. This minimizes the entry of water vapor from the outside air, thereby inhibiting microbial growth on the surface of the cherries. More importantly, it can effectively reduce water accumulation, thereby blocking the migration pathways of microorganisms [41].

### 3.5. The Study of Film Morphology

**Figure 3:**
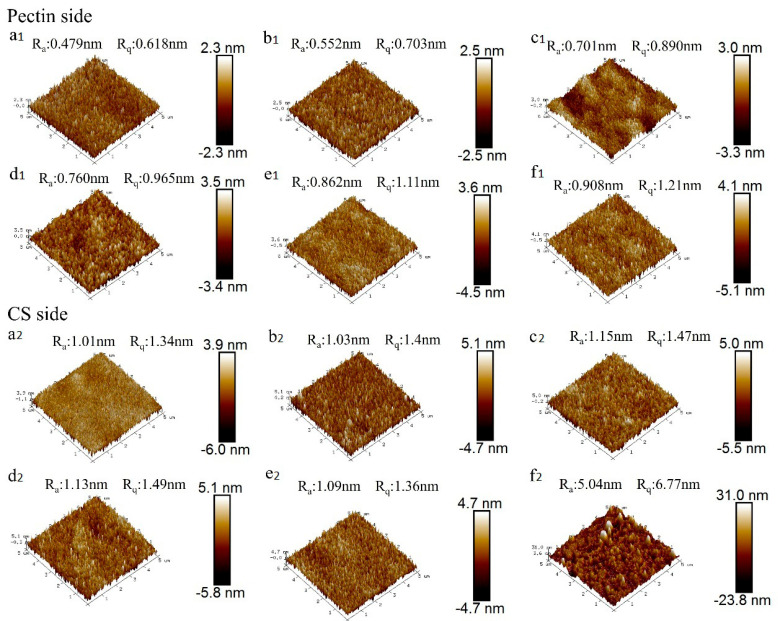
(**a1**–**f1**,**a2**–**f2**)Atomic force microscopy (AFM) images depicting the surface morphology of the pectin side and chitosan (CS) side of the bilayer films.

The surface roughness parameters Ra (arithmetic mean) and Rq (root mean square), measured via atomic force microscopy, quantitatively describe topographical variations in packaging materials. Engineered surface microstructures in food packaging films create precisely controlled functional barriers through optimized roughness profiles. As shown in the micrograph, the contrasting light and dark regions correspond to distinct elevation differences across the film surface. The Ra and Rq values of the CS film without lignin were 1.01 nm and 1.34 nm, respectively. The surface roughness analysis revealed distinct Ra = 1.03–5.04 nm and Rq = 1.40–6.77 nm values for lignin-incorporated CS membranes, demonstrating significant surface modification effects through lignocellulosic integration (Figure 3). Membranes with elevated lignin content exhibited engineered surface topography characterized by controlled roughness parameters that satisfy critical stability criteria for advanced food packaging applications [39]. The surface of the P/C film exhibits uniform characteristics, with an arithmetic mean roughness (Ra) of 0.479 nm and a root mean square roughness (Rq) of 0.618 nm. GSAE incorporation maintained stable roughness parameters despite lignin concentration variations, with the CS surface preserving planar integrity throughout modifications. These findings correlate with water contact angle measurements, confirming consistent surface hydrophilicity and morphological stability in lignocellulosic composite membranes. This corroborates findings by Rojo et al. [42] and Imani et al. [43]. Surface roughness is correlated with the hydrophobicity of the film, where larger roughness leads to stronger hydrophobicity. In previous studies, some research has enhanced the hydrophobicity of films by constructing large surface roughness [44,45].

### 3.6. Properties Related to Gas Permeability

**Figure 4:**
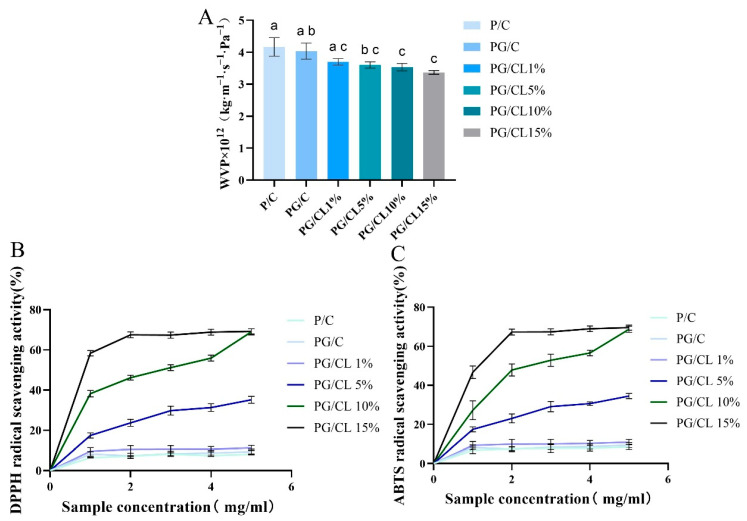
The evaluation of gas barrier properties (**A**) and antioxidant activity (**B**,**C**). Lowercase letters indicate significant differences in the properties of different films (*p* < 0.05).

Gas barrier performance fundamentally governs the effectiveness of active packaging systems in preserving cherries. The integration of GSAE into PG/C composite films shows a negligible impact on water vapor permeability (WVP) characteristics, with permeability values remaining comparable to baseline measurements (Figure 4A). However, as the concentration of lignin increases, the WVP value decreases to 3.41 (*p* < 0.05), indicating that a higher concentration of lignin enhances the water vapor barrier performance of the multi-active membrane. WVP is influenced by the hydrophilicity and hydrophobicity of the membrane, as well as the bioactive compounds added. The intermolecular forces within the membrane are crucial factors affecting its WVP. Lignin contains polar groups (-OH) that interact with the hydrophilic matrix of the membrane, creating a competitive binding effect [42]. This molecular interaction enhances mass transport resistance for water diffusion through the polymeric matrix, thereby reducing membrane WVP. The observed transport mechanism corresponds with established reports in barrier film research [32]. Active packaging films possess an optimal water vapor permeation barrier that helps to delay moisture loss from cherries. The presence of lignin introduces more tortuous paths within the film matrix, which serves as an explanation for the reduced water vapor diffusion process. This has also been validated in the work by Costa et al. [46,47].

### 3.7. Activity of Antioxidants of Films

The antioxidant function of the double-layer membrane is mediated through a free radical scavenging mechanism, which can be assessed by measuring the DPPH and ABTS radical scavenging rates. The results demonstrate that the P/C and PG/C membranes show scavenging rates of 8.16% and 9.32%, respectively, attributed to hydroxyl and amino groups within the natural polysaccharide network (Figure 4B,C). Upon lignin incorporation, the PG/CL membrane exhibits a concentration-dependent enhancement in antioxidant efficacy, achieving 67.51% DPPH scavenging at 2.0 mg/mL and 79.22% ABTS inhibition at 5.0 mg/mL. This performance improvement stems from the superior free radical scavenging capability of lignin’s polyphenolic architecture [8]. The experimental findings demonstrate that lignin-incorporated chitosan/pectin composite films exhibit enhanced antioxidative efficacy in controlling oxidative degradation processes during cherry preservation [48].

### 3.8. Crystalline Structure Analysis (XRD)

**Figure 5:**
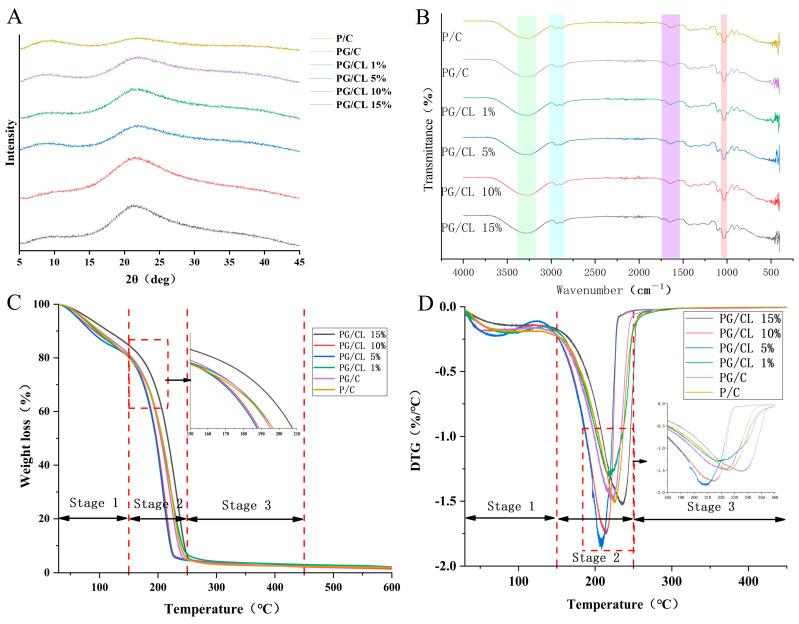
XRD (**A**), FT-IR spectra (**B**), and thermodynamic properties of bilayer films (**C**,**D**).

X-ray diffraction (XRD) analysis reveals that all the thin-film samples exhibit a broad amorphous peak near 2θ = 22°, indicative of an amorphous region and a semi-crystalline structure (Figure 5A). This finding is consistent with prior research, confirming the absence of distinct crystallites in the pectin phase, while chitosan (CS) exhibits a semi-crystalline state [32]. Comparative X-ray analysis indicates that lignin-doped films do not display significant shifts in peak positions or the emergence of new diffraction peaks relative to the PG/C control group, demonstrating excellent phase compatibility and hydrogen bond-mediated intermolecular interactions. As the lignin content progressively increases from 1% to 15%, the sharpness and intensity of the diffraction peaks concomitantly increase, which aligns with the increased hydrogen bonding between the phenolic hydroxyl groups of lignin and the amino groups of chitosan. Fourier transform infrared spectroscopy (FT-IR) corroborates this structural evolution through characteristic peak shifts at 3300–3500 cm^−1^ (O-H/N-H stretching vibrations) and 1650 cm^−1^ (C=O hydrogen bonds), validating that the polyphenol–polysaccharide interactions improve interfacial compatibility. The water vapor permeability (WVP) experiment further supports these findings [42].

### 3.9. Chemical Structure Characterization (FT-IR)

The molecular architecture of anthocyanin–lignin-modified pectin/chitosan hybrid composites was investigated through Fourier transform infrared spectroscopy (FT-IR) to elucidate their chemical bonding patterns and structural features. The stretching vibrations of the N-H bonds in chitosan and the O-H bonds within the polymer matrix were observed in the 3200–3500 cm^−1^ range, indicating the presence of polar interactions between membrane components (Figure 5B and Figures S1–S6). A distinct C-H stretching vibration band appeared at 2936 cm^−1^, while the characteristic absorption peak at 1738 cm^−1^ originated from the C=O vibrations of both esterified carboxyl groups (-COOR) and free carboxyl groups (-COOH) in pectin. Furthermore, the absorption band at 1653 cm^−1^ may arise from the stretching vibration of the C-O bonds in chitosan and asymmetric stretching vibrations of the carboxylate ions (COO−) in pectin [11]. Compared with the P/C control membrane, the PG/C membrane showed no significant shifts in hydrogen bond-related absorption peaks, demonstrating that anthocyanin addition does not affect the degree of hydroxyl association in the membrane. The observed changes in FT-IR spectra suggest that anthocyanin–lignin were integrated into the chitosan/pectin composite matrix through hydrogen bond-mediated intermolecular interactions [41].

### 3.10. Thermogravimetric Analysis (TGA)

The thermal stabilization effects of anthocyanin–lignin incorporation in pectin/chitosan (CS) composites were evaluated through TGA. Both TGA and derivative thermogravimetry (DTG) profiles exhibited three-stage decomposition kinetics: (1) 50–150 °C: mass loss from acetic acid/water evaporation with concurrent hydrogen bond dissociation (Figure 5C,D and Figure S7); (2) 150–250 °C: glycerol and bound water volatilization; (3) 250–450 °C: polymer backbone degradation. PG/CL15% demonstrated enhanced thermal stability due to lignin’s aromatic ether linkages and carbon–carbon bonds, which exhibit higher thermal decomposition thresholds compared with cellulose derivatives [34]. Consequently, the inclusion of more thermally stable lignin in PG/CL15% may provide significant advantages for composite membranes in specific applications. Between 350 and 450 °C, the decrease in the mass of the composite film gradually slows down, and the changes in the DTG curve also tend to stabilize, which proves that the existence of lignin enhances the thermal stability of the composite film.

### 3.11. Antimicrobial Properties of Films

**Figure 6:**
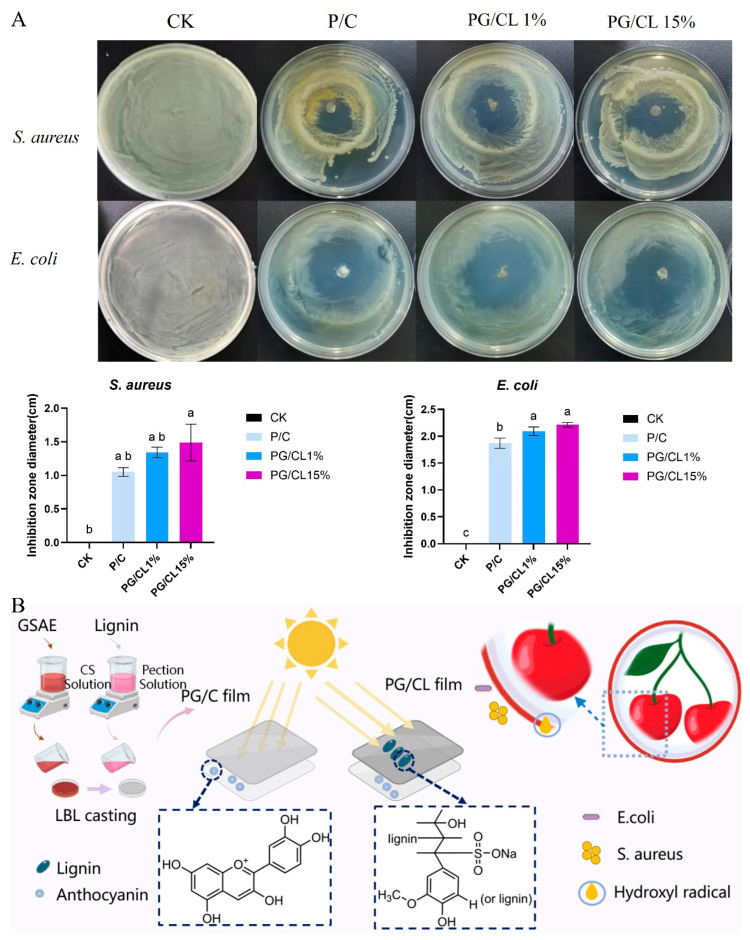
The antimicrobial characteristics and schematic representation of bilayer films are presented. The antibacterial properties are depicted in section (**A**), while the schematic illustration is provided in section (**B**). Lowercase letters indicate significant differences in the properties of different films (*p* < 0.05).

Cherry fruits are prone to contamination by foodborne pathogens and may spoil during storage and transportation, leading to significant economic losses and potential risks to human health. The experimental results show that the antibacterial effect of the P/C membrane against the two bacteria was limited (Figure 6A,B). This reduction in antibacterial efficacy is due to the fact that the antibacterial properties of chitosan depend on the positive charge groups it contains [49]. When lignin and anthocyanin were added to the membrane, it exhibited a strong inhibitory effect on *S. aureus* and *E. coli*, resulting in noticeable inhibition zones around the membrane. The antibacterial efficacy exhibited concentration-dependent enhancement, with the inhibition zone diameter gradually increasing at higher lignin concentrations. Notably, the PG/CL15% membrane demonstrated significantly larger inhibition zones against *S. aureus* and *E. coli* compared with the P/C and PG/C membranes, respectively (*p* < 0.05). This work establishes a scientific foundation for utilizing lignin-containing CS/pectin composites as multifunctional barriers against both pathogenic invasion and physiological decay in stored cherries. As the lignin concentration in the film increases, the antimicrobial activity of lignin-based films increases, which is attributed to the incorporation of additional active functional groups (e.g., aliphatic hydroxyl, carbonyl (C=O), and carboxyl (COOH)) into the film matrix. The disparities in antimicrobial efficacy can be rationalized by multiple factors: the Gram-staining characteristics of bacteria, exopolysaccharide synthesis capability, and variations in outer membrane permeability (which is inherently modulated by temperature), among others. Collectively, these factors, in conjunction with differential surface charges, may give rise to divergent antimicrobial activities against distinct bacterial species—this phenomenon also elucidates why the film exhibits inconsistent inhibitory effects on the two tested bacterial strains [50,51,52].

### 3.12. The Utilization of Multi-Active Packaging for the Preservation of Cherries

**Figure 7:**
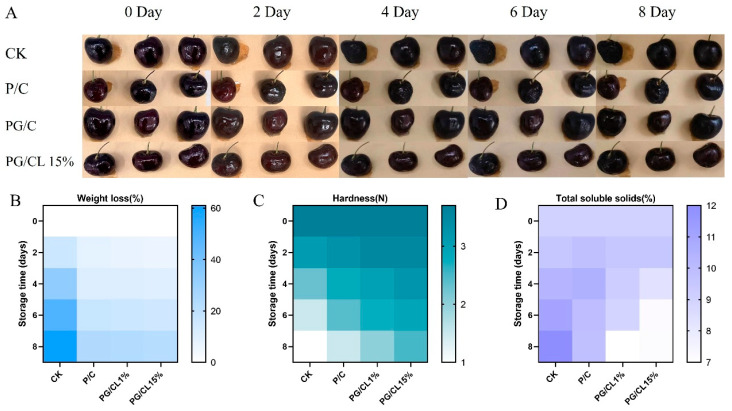
The quality attributes of cherries subjected to film-coating treatment include the following parameters: apparent quality of cherries (**A**), percentage of weight loss (**B**), hardness measured in Newtons (N) (**C**), and total soluble solids percentage (**D**).

To assess the preservation capabilities of the film, cherries were immersed in the film solution and air-dried to evaluate the extent of decay. As time went by, the cherries exhibited varying degrees of dehydration and decay. After 4 days of storage without packaging treatment in the control group, the cherries showed signs of decay. In contrast, the PG/C and PG/CL15% packaging films kept the cherries fresh for up to 6 days. Notably, cherries treated with PG/CL15% remained free from infection throughout the entire storage period (Figure 7A). This antibacterial effect is attributed to the antibacterial properties of anthocyanins present in the packaging film. Additionally, the lignin in the PG/CL15% coating serves as a UV shield, effectively protecting the anthocyanins and preventing significant loss due to photodegradation (Figure 7B). The film’s hydrophilic and hydrophobic characteristics allow the water vapor produced by the cherries to be retained within the coating material, thereby delaying the respiration process of the cherries [30]. The analysis revealed that cherries coated with PG/CL15% demonstrated superior texture preservation, showing a firmness loss rate of 32.43% after 8-day storage compared with 45.2–52.8% in the control groups (Figure 7C). This study indicated an inverse trend in soluble solids content (SSC) variation: the uncoated control group exhibited an 18.6% SSC increase, while the coated samples maintained stable SSC levels through the gas barrier-mediated regulation of respiratory activity (Figure 7D). The coating treatment established a microenvironment with elevated carbon dioxide (CO_2_) and reduced oxygen (O_2_) levels within the film. This atmospheric composition suppressed respiratory activity in cherries, consequently delaying the accumulation of secondary metabolites such as anthocyanins and phenolic compounds [53]. Compared with the coatings used in previous studies on cherries, strawberries, and tomatoes, the film in this study demonstrates better advantages in extending shelf life, exhibits stronger antibacterial efficacy against common spoilage microorganisms, and features simpler preparation and application processes, thereby highlighting its significant practical application potential in the field of food preservation. Although these films help preserve cherries, the impact of the films on the fruit’s flavor and other sensory characteristics is also of great importance. Currently, research on these effects is insufficient and requires further in-depth study in the future.

## 4. Conclusions

This study establishes a distinctive bilayer packaging approach by strategically incorporating grape seed extract (GSAE) and lignin into separate layers of a pectin/chitosan matrix—contrasting with conventional single-layer composites that homogeneously blend functional additives. The optimized PG/CL bilayer film (15% *w*/*w* lignin) demonstrated significant enhancements in UV blocking, mechanical strength, gas barrier properties, and antioxidant capacity, validated through comprehensive characterization. Crucially, its dual-functional design includes (1) an outer lignin layer for UV shielding and antibacterial barriers and (2) an inner GSAE layer for sustained antibacterial release. Preservation tests confirm its efficacy for cherry shelf-life extension. Beyond cherries, this scalable bilayer strategy offers a promising solution to reduce food waste in perishable produce, leveraging renewable biomaterials. Future work should validate commercial adaptability across diverse climacteric fruits.

## Figures and Tables

**Table 1:** The characteristics of color parameters, opacity, and physical appearance of composite films.

Film	Color					Opacity (mm^−1^)	Physical Appearance
	L	a	b	ΔE	WI		
P/C	34.15	−0.15	0.70	59.3	34.1	0.22	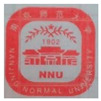
PG/C	32.59	0.04	1.32	60.9	32.6	0.35	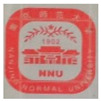
PG/CL1%	32.88	0	1.46	60.6	32.9	0.39	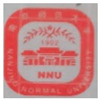
PG/CL5%	32.59	0.05	1.68	60.9	32.6	0.53	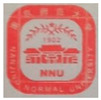
PG/CL10%	33.83	−0.03	1.05	59.7	33.8	0.63	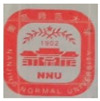
PG/CL15%	33.37	−0.04	1.71	60.1	33.3	0.77	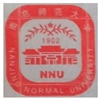

Values are the average of results measured on three films of the same lot.

## Data Availability

The original contributions presented in this study are included in the article/Supplementary Materials. Further inquiries can be directed to the corresponding authors.

## Supplementary Materials

The following supporting information can be downloaded at: https://www.mdpi.com/article/10.3390/foods14132283/s1. Figure S1. The FT-IR image of a P/C film. Figure S2. The FT-IR image of a PG/C film. Figure S3. The FT-IR image of a PG/CL1% film. Figure S4. The FT-IR image of a PG/CL5% film. Figure S5. The FT-IR image of a PG/CL10% film. Figure S6. The FT-IR image of a PG/CL15% film. Figure S7. The thermogravimetric loss rate of the film between 150 °C and 210 °C (A). The DTG curve of the film between 180 °C and 260 °C (B).
