# Supplementary material for: Development of Double-Film Composite Food Packaging with UV Protection and Microbial Protection for Cherry Preservation
Source: Foods. 2025 Jun 27;14(13):2283. doi: 10.3390/foods14132283 (PMC12249171; doi:10.3390/foods14132283)
Supplement: Supplementary file 1 [file foods-14-02283-s001.zip › foods-3695093-supplementary.pdf]

Development of Double-Film Composite Food Packaging with UV Protection and Microbial Protection for Cherry Preservation

Han Wang [1], Yanjing Liao [1], Guida Zhu [1], Longwen Wang [1], Zihan Chen [1], Xue Li [1], Chao Wang [2], Jing Yu [1,*] and Ping Song [1,*]

[1] School of Food Science and Pharmaceutical Engineering, Nanjing Normal University, Nanjing 210023, China
[2] Science Center for Future Food, Jiangnan University, Wuxi 214122, China
* Correspondence: author: jingyu@njnu.edu.cn (J.Y.); 77010@nnu.edu.cn (P.S.); Tel./Fax: +86-25-85898687 (J.Y. & P.S.)

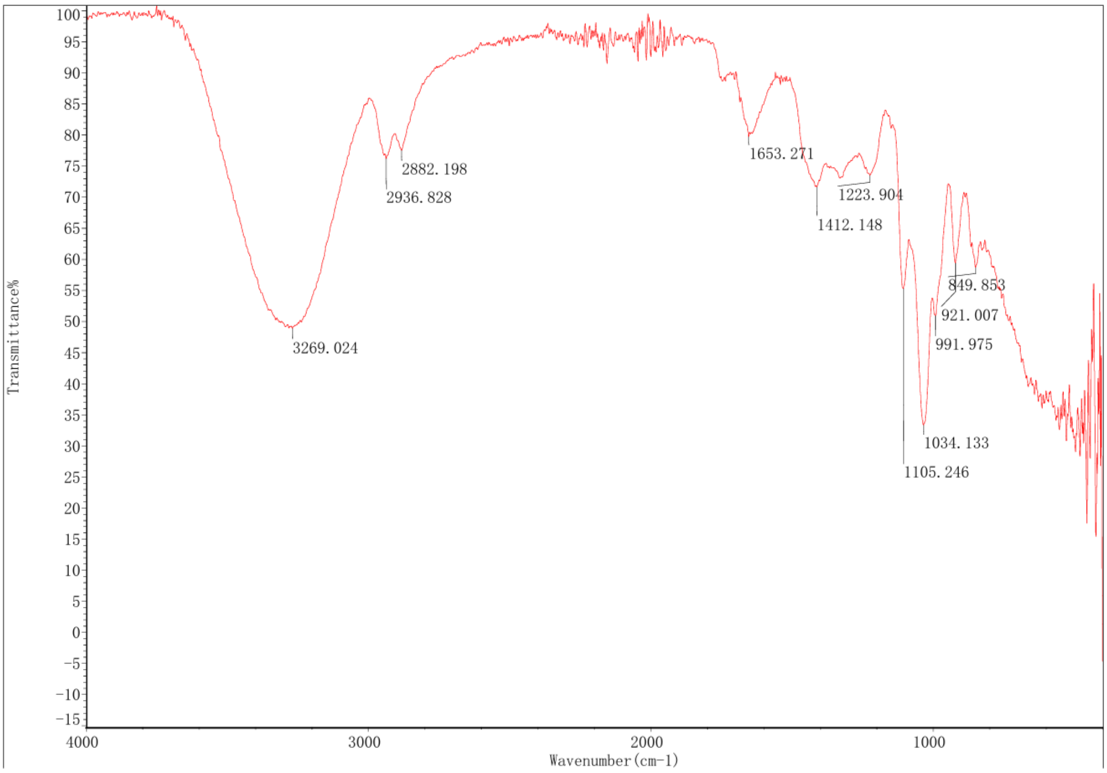

Figure S1. The FT-IR image of a P/C film.

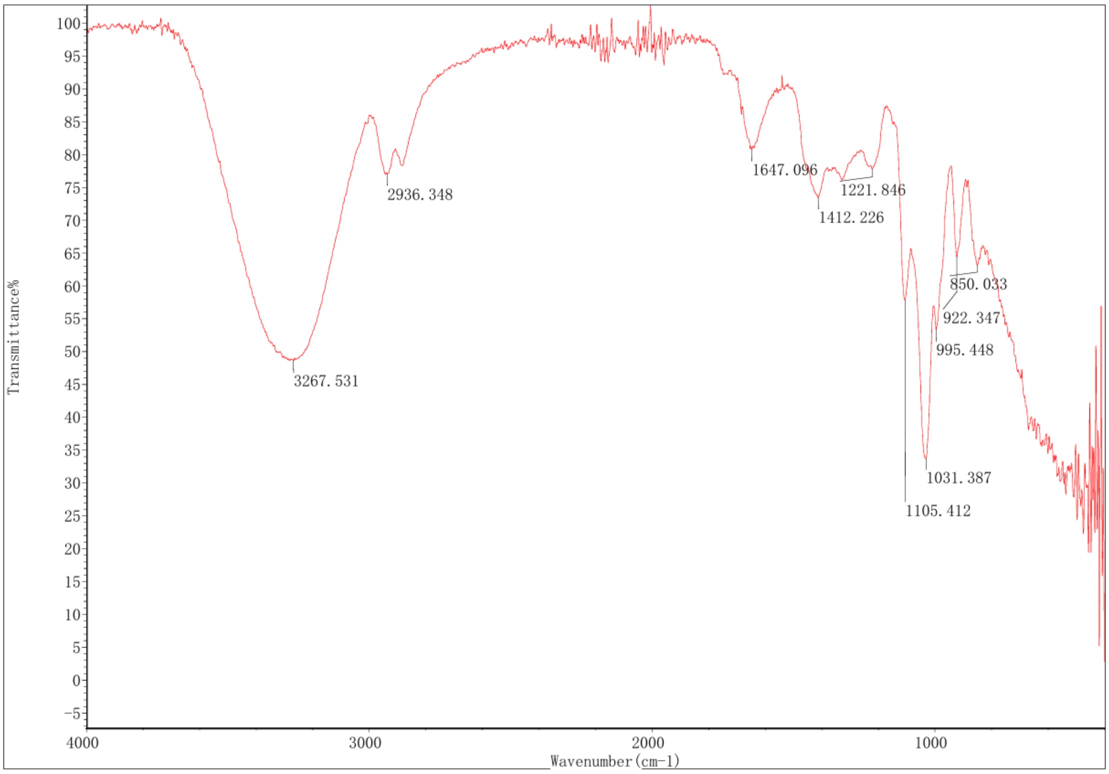

Figure S2. The FT-IR image of a PG/C film.

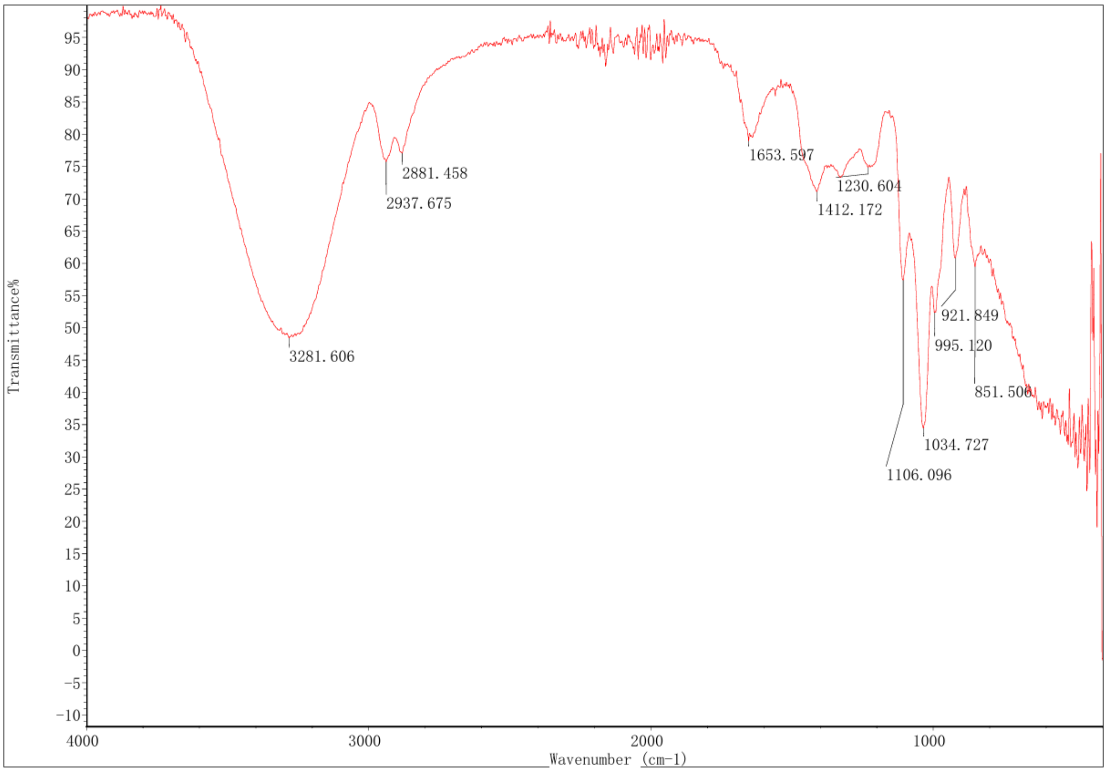

Figure S3. The FT-IR image of a PG/CL1% film.

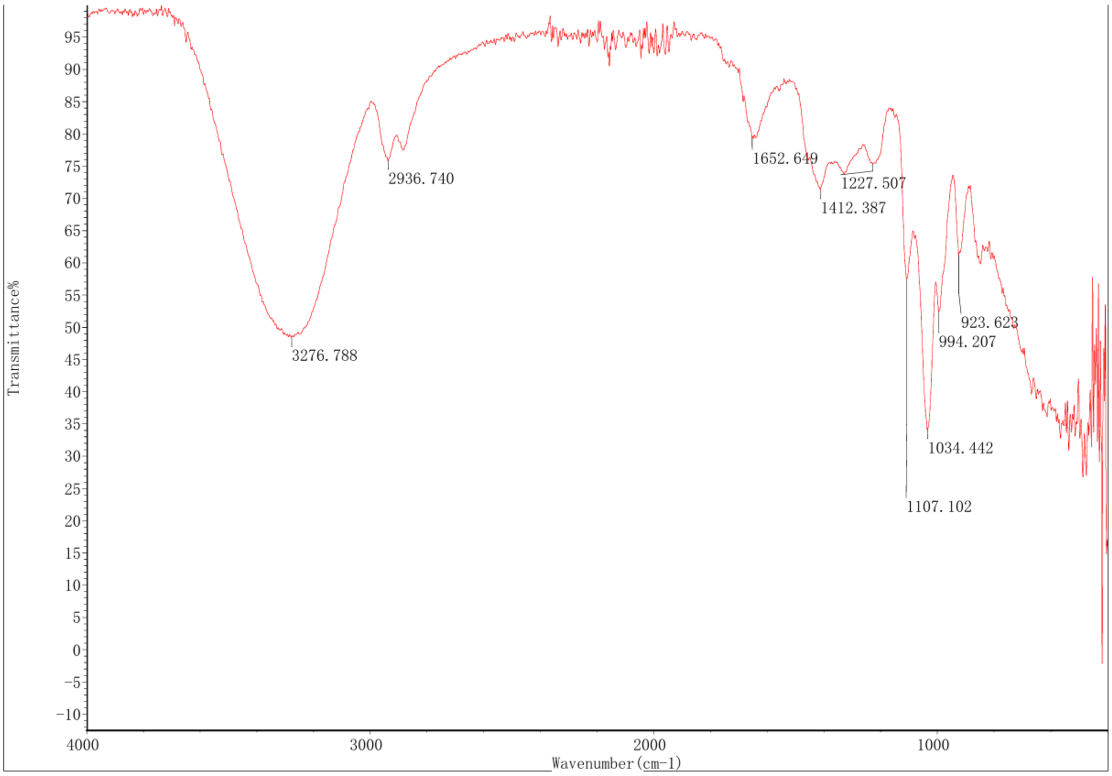

Figure S4. The FT-IR image of a PG/CL5% film.

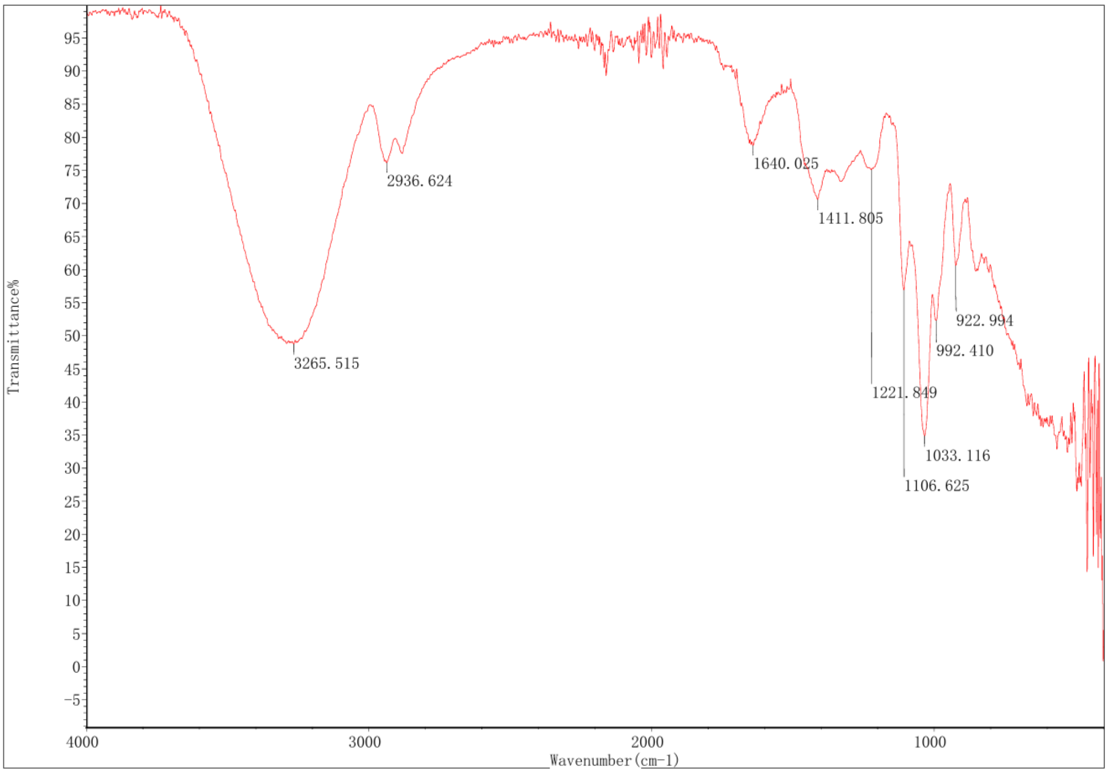

Figure S5. The FT-IR image of a PG/CL10% film.

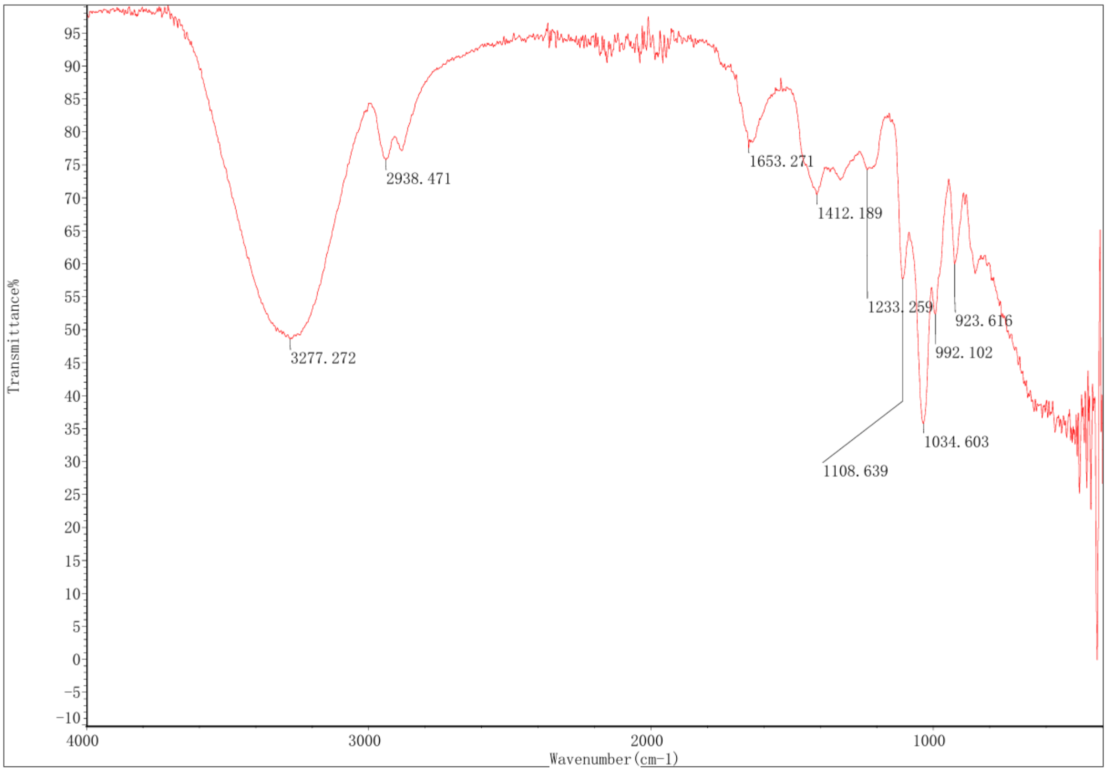

Figure S6. The FT-IR image of a PG/CL15% film.

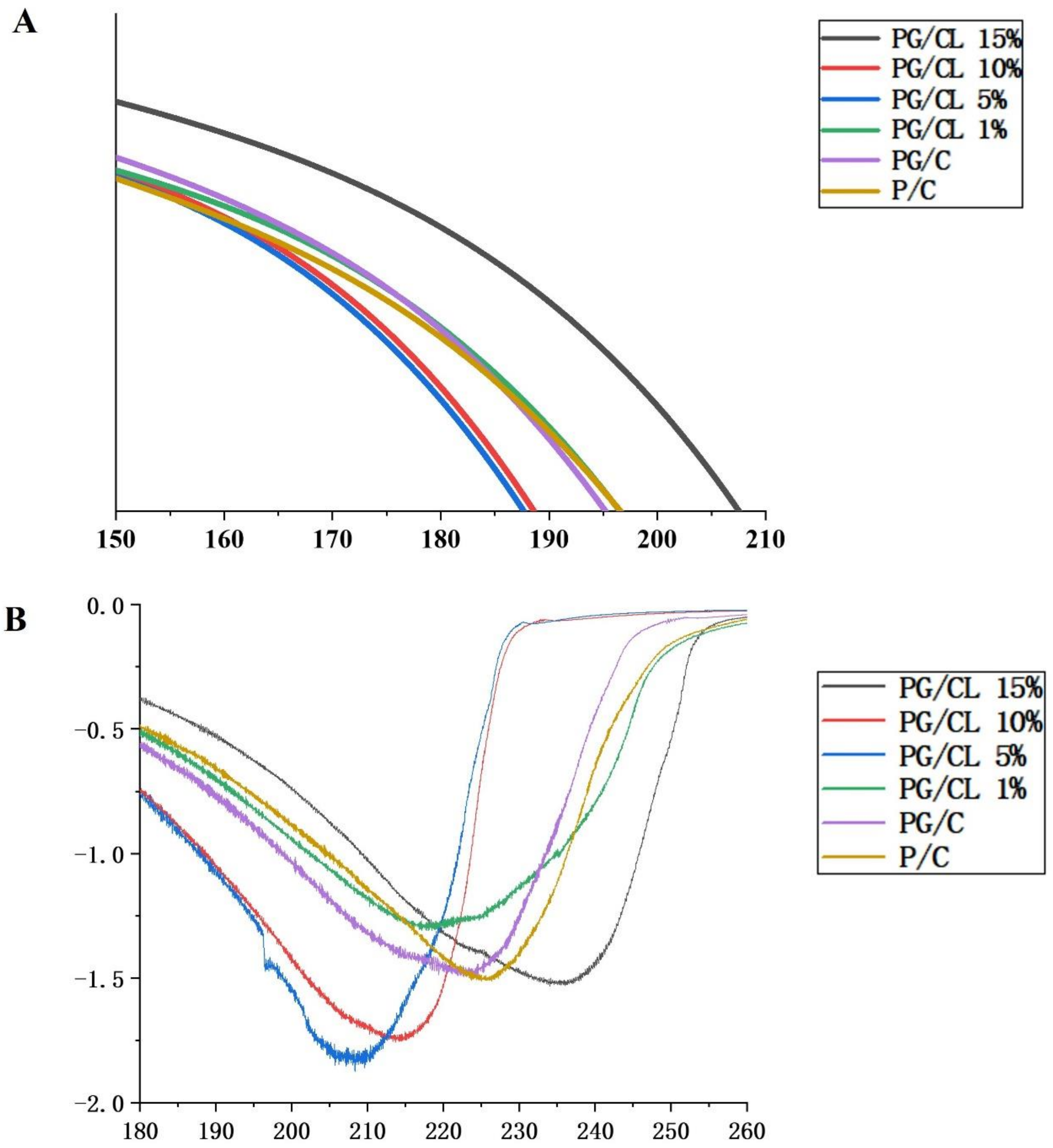

Figure S7. The thermogravimetric loss rate of the film between 150°C and 210°C(A).

The DTG curve of the film between 180°C and 260°C(B).
